# Decorating sdAbs with Chelators: Effect of Conjugation on Biodistribution and Functionality

**DOI:** 10.3390/ph14050407

**Published:** 2021-04-25

**Authors:** Henri Baudhuin, Janik Puttemans, Heleen Hanssens, Philippe Vanwolleghem, Sophie Hernot, Geert Raes, Catarina Xavier, Tony Lahoutte, Pieterjan Debie

**Affiliations:** 1 Department of Medical Imaging (MIMA), Vrije Universiteit Brussel, Laarbeeklaan 103, B-1090 Brussels, Belgium; Janik.Puttemans@vub.be (J.P.); Heleen.Hanssens@vub.be (H.H.); philippe.vanwolleghem@ppms.be (P.V.); Sophie.Hernot@vub.be (S.H.); Catarina.Xavier@vub.be (C.X.); Tony.Lahoutte@uzbrussel.be (T.L.); Pieterjan.Debie@vub.be (P.D.); 2 Unit of Cellular and Molecular Immunology (CMIM), Vrije Universiteit Brussel, Pleinlaan 2, B-1050 Brussels, Belgium; Geert.Raes@vub.be; 3 Myeloid Cell Immunology Laboratory, VIB Center for Inflammation Research, Pleinlaan 2, B-1050 Brussels, Belgium; 4 Nuclear Medicine Department (NUCG), Universitair Ziekenhuis Brussel (UZ Brussel), Laarbeeklaan 101, B-1090 Brussels, Belgium

**Keywords:** bioconjugation, ion exchange chromatography, molecular imaging, radiopharmaceutical, tracer optimization, kidney clearance

## Abstract

Single domain antibodies (sdAbs) have proven to be valuable probes for molecular imaging. In order to produce such probes, one strategy is the functionalization of the reactive amine side chain of lysines with a chelator, resulting in a mixture of compounds with a different degree of conjugation. In this study, we implemented anion exchange chromatography (AEX) to separate the different compounds or fractions that were further characterized and evaluated to study the impact of the conjugation degree on pharmacokinetic properties and functionality. Anti-HER2 and anti-MMR sdAbs were functionalized with NOTA or DTPA chelator. Anion exchange chromatography was performed using 0.02 mol/L Tris pH 7.5 as the first solvent and 0.25 M or 0.4 M NaCl (in case of NOTA chelator or DTPA chelator, respectively) as the second solvent applied as a gradient. The fractions were characterized via mass spectrometry (MS), surface plasmon resonance (SPR), and isoelectric focusing gel electrophoresis (IEF), while in vivo studies were performed after radiolabeling with either ^68^Ga (NOTA) or ^111^In (DTPA) to assess the impact of the conjugation degree on pharmacokinetics. AEX could successfully be applied to separate fractions of (chelator)_n_-anti-HER2 and (chelator)_n_-anti-MMR sdAb constructs. MS confirmed the identity of different peaks obtained in the separation process. SPR measurement suggests a small loss of affinity for (chelator)_3_-anti-sdAb, while IEF revealed a correlated decrease in isoelectric point (pI) with the number of conjugated chelators. Interestingly, both the reduction in affinity and in pI was stronger with the DTPA chelator than with NOTA for both sdAbs. In vivo data showed no significant differences in organ uptake for any construct, except for (DTPA)_n_-anti-MMR, which showed a significantly higher liver uptake for (DTPA)_1_-anti-MMR compared to (DTPA)_2_-anti-MMR and (DTPA)_3_-anti-MMR. For all constructs in general, high kidney uptake was observed, due to the typical renal clearance of sdAb-based tracers. The kidney uptake showed significant differences between fractions of a same construct and indicates that a higher conjugation degree improves kidney clearance. AEX allows the separation of sdAbs with a different degree of conjugation and provides the opportunity to further characterize individual fractions. The conjugation of a chelator to sdAbs can alter some properties of the tracers, such as pI; however, the impact on the general biodistribution profile and tumor targeting was minimal.

## 1. Introduction

Nuclear molecular imaging can be used for cancer diagnosis, staging, and characterization. Although [^18^F]FDG is still the gold standard PET tracer, more and more targeted tracers are being developed that are specifically directed against particular cancer markers.

Due to the ease of their generation and their wide availability, monoclonal antibodies (mAbs) were the basis for the design of several target-specific tracers [1]. However, due to their long blood retention—caused by their large size and the presence of an intact Fc-region, the optimal imaging timepoint for mAb-based tracers is typically several days after administration [2]. This requires the use of long-lived radionuclides and leads to complex logistics and non-negligible radiation doses to healthy tissues in patients [3]. Therefore, there has been a strong focus on the development of novel tracer molecules with faster kinetics that allow an earlier imaging timepoint, reducing the radiation dose to healthy tissues and improving the overall imaging contrast. Antibody fragments [4], scaffold proteins [5], and peptides [6] have all been shown to be capable of this.

One particular targeting vehicle of interest is the single-domain antibodies (sdAbs), which are also called VHHs or Nanobodies. SdAbs are the smallest antigen-binding domain usually derived from heavy chain-only antibodies found in Camelidea [7] and can be generated against virtually any target via, for example, in vivo immunization [8]. Due to their natural occurrence without a corresponding light chain, sdAbs are stable in aqueous solutions, and they retain a high binding affinity despite only possessing three complementarity-determining regions [9]. Additionally, sdAbs are small molecules (≈15 kDa), rapidly cleared via the renal system, with appropriate pharmacokinetics for imaging, which makes them attractive as a platform for the design of nuclear imaging tracers [10,11,12,13]. A variety of sdAb-based imaging tracers have been developed for imaging in oncology [14,15,16,17,18], cardiovascular diseases [19,20,21], and inflammation [22]. Beyond preclinical development, two sdAbs are currently being investigated as PET tracers in clinical trials. The first tracer, targeting the human epidermal growth factor receptor 2 (HER2), [^68^Ga]Ga-NOTA-anti-HER2 sdAb, is being investigated in two clinical studies for (1) diagnosis of HER2+ breast cancer [23] (NCT03924466) and (2) detection of HER2+ brain metastasis (EudraCT 2015-002328-24/NCT03331601), while the second tracer, targeting the macrophage mannose receptor (MMR), [^68^Ga]Ga-NOTA-anti-MMR sdAb, is being investigated in a Phase I/II study (EudraCT 2017-001471-23), to image and detect MMR+ tumor-associated macrophages [24], in order to predict treatment responses based on the MMR+ macrophage content.

The development of radiolabeled tracers for clinical applications in both imaging and therapy entails proper characterization and documentation of the product. MAbs are known to provide a range of variants upon conjugation with any effector molecule, as the conjugation is performed on the residue of a certain amino acid, typically the primary amine side chain of lysines [25]. Since they are relatively large proteins, antibodies contain many different lysines in their structure [25], making the conjugation an uncontrolled process for both the number of conjugated effector molecules as well as for the site of conjugation [26]. Random conjugation on lysine residues of antibodies has been reported to generate over one million different species [27]. As such, in the case of conjugation with a chelator, a heterogenous mixture of conjugated mAbs with different chelator-to-protein ratios (C/P) and conjugated mAbs with a same C/P ratio but attached at different sites (regio-isomers) is obtained. In turn, this variety in conjugation can lead to important differences in properties between the different variants, such as pI, hydrophobicity, solubility, and antigen-binding capacity [28,29,30]. These differences in properties can lead to important differences in parameters such as pharmacokinetics, toxicity, and functionality [26,31,32]. Indeed, it has been shown that a higher C/P ratio can lead to for example increased liver uptake, increased spleen uptake, and/or reduced tumor uptake [33,34,35]. Therefore, the degree of conjugation should be carefully evaluated. For antibody-based tracers, the number of obtained variants upon conjugation (different C/P, without taking regio-isomers into account) is high, and investigating each fraction for its biodistribution and functionality is cumbersome.

However, single-domain antibody-based tracers are much smaller (about 10 times) and therefore possess far fewer lysines. Consequently, the number of possible conjugated chelators per sdAb is limited, and investigating sdAb-variants with different C/P ratios becomes feasible.

Although the tracers discussed in this manuscript are not conjugated with a cytotoxic payload, the general rules for the characterization of antibody-based compounds or fractions thereof still apply. In that regard, we undertook steps to evaluate the impact of the chelator conjugation degree and to characterize each obtained fraction (with different C/P) individually. Obtaining this information on each fraction separately not only accounts for a more complete medical dossier but also allows a more thorough risk and safety assessment and could facilitate the process of registering a new Investigational Medicine Product (IMP).

Therefore, with the intention of providing a better characterization of the clinically relevant sdAb-based tracers anti-HER2 (2Rs15d) [23,36,37] and anti-MMR (MMR3.49) [14,24], we aimed to investigate the effect of different C/P ratios of these two sdAbs, which are conjugated with NOTA for ^68^Ga-labeling or DTPA for ^111^In-labeling. This was achieved by using AEX, which allows the separation of differently conjugated sdAb-chelator fractions, followed by in vitro and in vivo assessment of each fraction.

## 2. Results

### 2.1. AEX and Product Characterization

**Figure 1:**
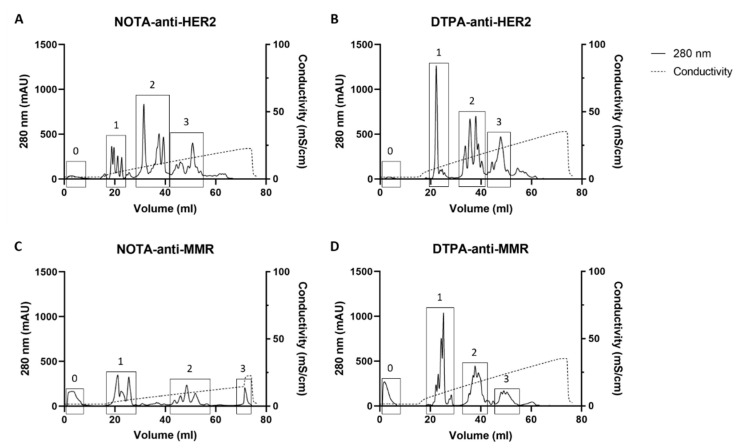
AEX purification profiles of (**A**) NOTA-anti-HER2, (**B**) DTPA-anti-HER2, (**C**) NOTA-anti-MMR, and (**D**) DTPA-anti-MMR. Clusters of peaks containing (chelator)_1_-sdAb (fraction 1), (chelator)_2_-sdAb (fraction 2), or (chelator)_3_-sdAb (fraction 3) are framed denoted with 1, 2, and 3, respectively. Frames denoted with 0 correspond to unconjugated sdAb. The separation of fractions is achieved by an increasing salt gradient resulting in increasing conductivity. The clusters or fractions were defined by identification of each peak individually via MS.

After purification of the conjugated sdAb via SEC, the collected product was further subjected to AEX, which was able to separate various subpopulations present in the conjugated samples (Figure 1). Clusters of peaks could be determined, which contained either (chelator)1-sdAb (fraction 1, F1), (chelator)2-sdAb (fraction 2, F2), or (chelator)3-sdAb (fraction 3, F3), and within one cluster (or fraction), we identified amidated and deamidated forms, which have a different pI and already count for at least two peaks per cluster. Additionally, the presence of the other peaks within one fraction is likely a result of slightly altered column interactions by regio-isomers.

**Figure 2:**
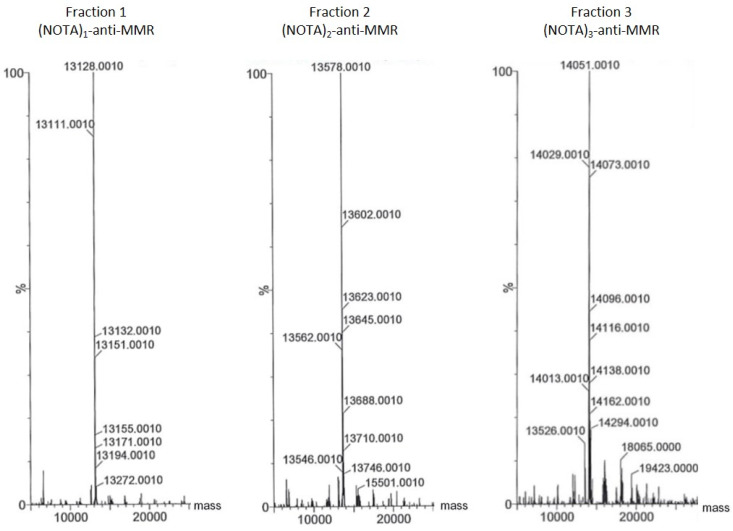
Mass spectrometry spectra of (NOTA)1-anti-MMR (fraction 1), (NOTA)2-anti-MMR (fraction 2), and (NOTA)3-anti-MMR (fraction 3). Theoretical molecular weights were calculated at 13,128.6, 13,579.1, and 14,029.6, respectively. Mass measurement error is 1 Da.

Mass spectrometry was used to confirm the identity of the fractions upon purification as exemplified for the NOTA-MMR construct (Figure 2). On the spectrum data, for fraction 1, two main peaks with molecular weight 13,128 and 13,111, σ = 1 Da were observed, corresponding to the expected mass [M] of (NOTA)1-anti-MMR (theoretical MW = 13,128.6) and deamidated compound [M-17]. On fraction 2, three main peaks were observed, 13,602, 13,578, 13,562, σ = 1 Da, corresponding to [M-17], [M] and [M+Na+], and confirming the presence of (NOTA)2-anti-MMR (theoretical MW = 13,579.1 Da). For fraction 3, main peaks were observed at 14,029, 14,051, and 14,073, σ = 1 Da, corresponding to [M], [M+Na+], and [M+2Na+], confirming that the fraction corresponds to (NOTA)3-anti-MMR (theoretical MW = 14,029.6). The corresponding deamidated form is present as a minor peak at 14,013. The full spectra of each construct for both the anti-HER2 and anti-MMR sdAb are available in supplemental data (Supplemental Figures S1–S4).

For each construct, the affinity was evaluated via SPR (Table 1). The affinity (a measurement strength of interaction) is represented as equilibrium dissociation constant KD (ratio of koff/kon), where a lower KD is correlated with a higher affinity and vice versa. It seems from this measurement that the conjugation degree can influence the affinity, most notably on fraction 3.

An additional characterization of the fractions was performed via IEF electrophoresis, which allows measurement of the pI of each fraction (Table 2).

Unconjugated sdAbs (reference) show a pI above the upper limit of the gel of 8.3, while a strong decrease in pI was observed upon the conjugation of a chelator. The pI further decreased as the number of chelators increased. Additionally, the pI of DTPA-conjugated sdAbs is lower than the NOTA-counterpart. For each fraction, two or more bands were observed, corresponding to intact or deamidated form and potentially due to regio-isomers having a small difference in pI or a different interaction with the gel. An important limitation of the IEF electrophoresis is the lower limit of pI 3.5, meaning that all measured pI’s below 3.5 are estimations calculated by the gel analysis software. An example of an IEF gel is available in supplemental data (Supplemental Figure S5).

### 2.2. In Vivo Biodistribution

In vivo biodistribution studies were performed for each construct, where the fractions were injected individually in tumor-bearing mouse models to assess any difference in in vivo behavior. An additional study was performed for the NOTA-anti-MMR in a MMR KO model for a specific assessment on kidney uptake. Average uptake values per organ are available for each study in supplementary data (Supplemental Tables S5–S9).

#### 2.2.1. [^68^Ga]Ga-NOTA-anti-HER2

**Figure 3:**
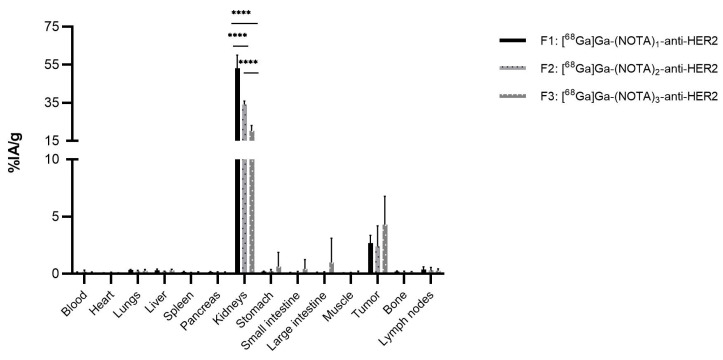
Ex vivo biodistribution analysis of [^68^Ga]Ga-(NOTA)_n_-anti-HER2 fractions in athymic nude mice (*n* = 6 per group), with a human HER2 expressing SKOV-3 tumor. (**** *p* < 0.001).

The biodistribution of [^68^Ga]Ga-(NOTA)_n_-anti-HER2 shows a background signal (%IA/g ≤ 0.5%) in most organs and tissues, with a notable exception of the kidneys and tumor (Figure 3). The low background in most organs was expected, as the anti-HER2 sdAb is specific to human HER2 protein. The high kidney uptake is typical for sdAb-based tracers, as these tracers are filtered and cleared via the kidneys, with partial uptake of the tracer due to the protein reuptake mechanism in proximal tubuli.

Kidney uptake values of 53.1%IA/g, σ = 6.4% (F1), 34.2%IA/g, σ = 1.7% (F2), and 20.2%IA/g, σ = 3.0% (F3) were obtained, respectively, with F1 showing a significantly higher uptake compared to F2 and F3, and F2 showing a significantly higher uptake than F3 (all *p* < 0.001). Tumor uptake values of 2.7%IA/g, σ = 0.6% (F1), 2.4%IA/g, σ = 1.6% (F2), and 4.3%IA/g, σ = 2.1% (F3) were obtained, respectively. Previous specificity studies [36,38] support the specific uptake of the fractions in the tumors, while the signal in stomach and large intestine of F3 is likely due to the contamination of samples during experiment.

#### 2.2.2. [^111^In]In-DTPA-anti-HER2

**Figure 4:**
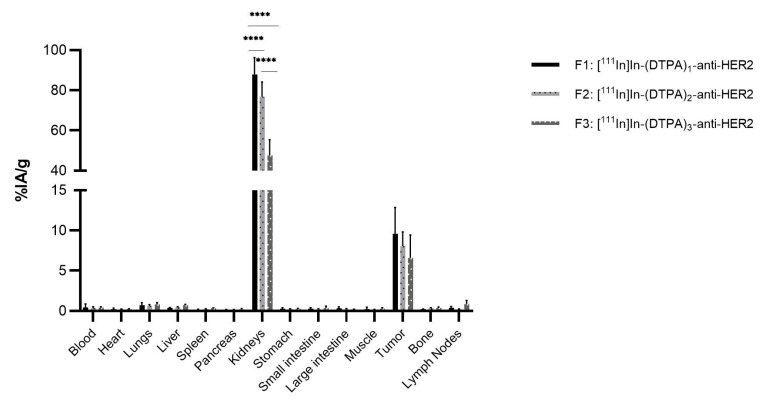
Ex vivo biodistribution analysis of [^111^In]In-(DTPA)_n_-anti-HER2 fractions in athymic nude mice (*n* = 6 per group), with a human HER2 expressing SKOV-3 tumor (**** *p* < 0.001).

Similarly to the NOTA-anti-HER2 study, biodistribution of [^111^In]In-(DTPA)_n_-anti-HER2 fractions was assessed in athymic nude mice with human HER2-expressing tumors (Figure 4).

As expected, a background signal (%IA/g ≤ 0.5%) is observable for most organs and tissues, except for the kidneys and tumor, which shows a clear uptake. Kidney uptake values of 87.9%IA/g (σ = 8.4%) (F1), 77.0%IA/g (σ = 7.2%) (F2), and 48.0%IA/g (σ = 6.2%) (F3) were obtained, respectively. Similarly, as the previous study, F1 showed a significantly higher kidney uptake compared to F2 and F3, while F2 showed a significantly higher uptake compared to F3 (all *p* < 0.001). The kidney uptake pattern is consistent with the previous observation, where it seems that a higher degree of conjugation is associated with reduced kidney uptake. Tumor uptake values of 9.6%IA/g, σ = 3.4% (F1), 8.1%IA/g, σ = 1.8% (F2), and 6.6%IA/g, σ = 2.8% (F3) were obtained, respectively.

**Figure 5:**
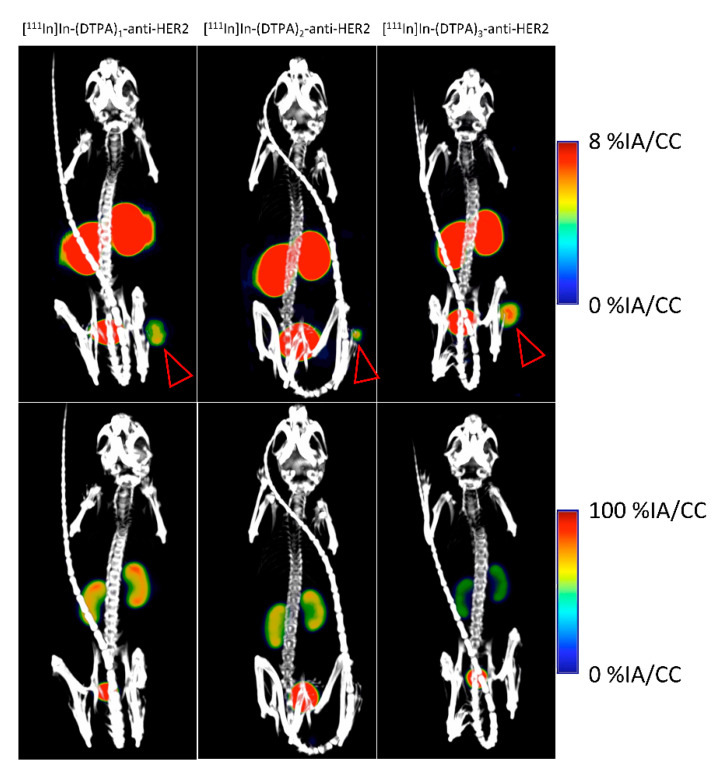
SPECT/CT imaging of different [^111^In]In-(DTPA)_n_-anti-HER2 fractions 1 h p.i. in SKOV3 tumor-bearing athymic nude mice. Scaling was adjusted depending on the tracer uptake values of the organ in focus. The (**upper panels**) are focused on the tumor (denoted by a red arrow). The (**lower panels**) are focused on the kidneys, which show a decrease in signal with increasing chelator substitution.

SPECT/CT images of [^111^In]In-(DTPA)n-anti-HER2 fractions show a clear tumor uptake for all three fractions (Figure 5—Upper panel), while the kidney signal clearly reduces for F2 and even more for F3 (Figure 5—Lower panel).

#### 2.2.3. [^68^Ga]Ga-NOTA-anti-MMR

**Figure 6:**
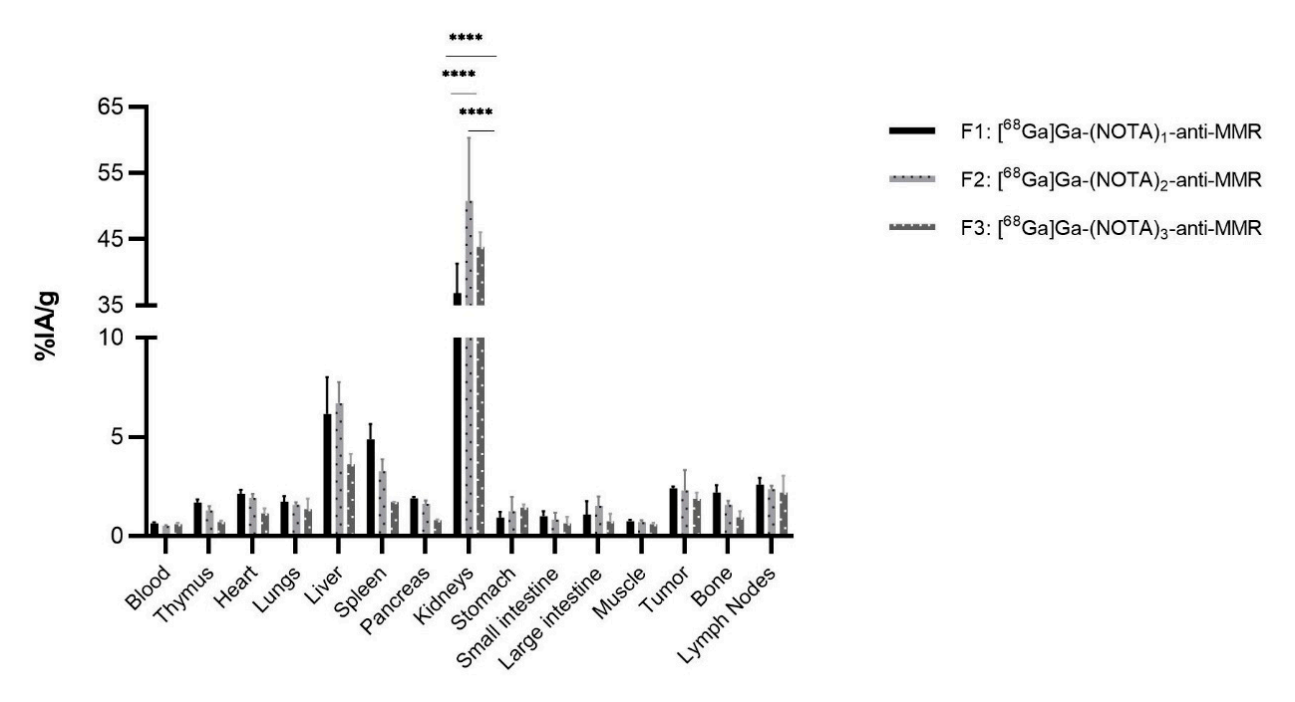
Ex vivo biodistributions analysis of [^68^Ga]Ga-(NOTA)_n_-anti-MMR fractions in C57Bl/6 mice (*n* = 3 per group), with a 3-LLR tumor (**** *p* < 0.001).

The biodistribution of [^68^Ga]Ga-(NOTA)_n_-anti-MMR fractions shows uptake in several organs, such as thymus, heart, lungs, liver, spleen, pancreas, bone, and lymph nodes (Figure 6), as expected, as the anti-MMR sdAb is cross-reactive for murine and human MMR.

Tumor uptake values of, respectively, 2.4%IA/g, σ = 0.1% (F1), 2.3%IA/g, σ = 1.0% (F2), and 1.9%IA/g, σ = 0.3% (F3) were obtained. The kidney uptake shows a different pattern compared to the anti-HER2 constructs, with uptake values of 36.9%IA/g, σ = 4.4% (F1), 50.8%IA/g, σ = 9.5% (F2), and 43.9%IA/g, σ = 2.2% (F3), respectively. Here, F1 showed a significantly lower kidney uptake compared to F2 and F3 (*p* < 0.001), while F2 shows a significantly higher kidney uptake compared to F3 (*p* < 0.001). Despite the omission of one cohort from analysis, a sample size of 3 mice per group was still sufficient to measure significant differences between the kidney uptake of the different fractions.

#### 2.2.4. [^111^In]In-DTPA-anti-MMR

**Figure 7:**
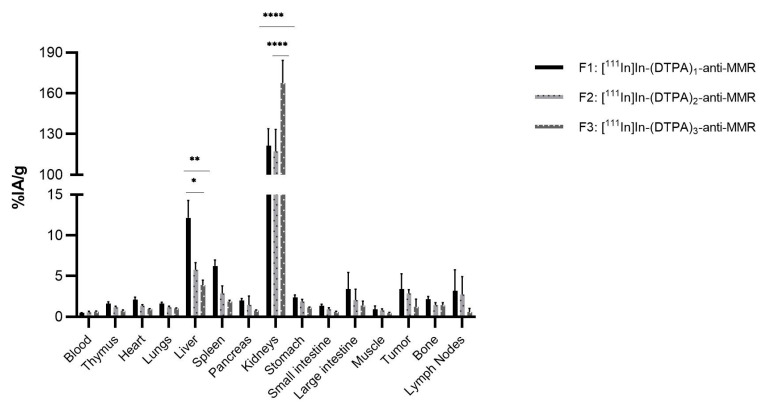
Ex vivo biodistribution analysis of [^111^In]In-(DTPA)_n_-anti-MMR fractions in C57Bl/6 mice (*n* = 6 per group), with a resistant LLC tumor variant. (* *p* < 0.05, ** *p* < 0.01, **** *p* < 0.001).

The biodistribution of [111In]In-(DTPA)_n_-anti-MMR fractions shows, next to tumor and kidney uptake, uptake in several organs as well, as expected (Figure 7). The liver showed uptake values of 12.2%IA/g, σ = 2.1%) (F1), 5.8%IA/g, σ = 0.9% (F2), and 3.9%IA/g, σ = 0.6% (F3), respectively, with F1 being significantly higher than F2 (*p* < 0.05) and F3 (*p* < 0.01). Tumor uptake values of 3.4%IA/g, σ = 1.9% (F1), 2.9%IA/g, σ = 0.5% (F2), and 1.2%IA/g, σ = 0.9% (F3) were obtained, while the kidneys showed an uptake of 121.4%IA/g, σ = 12.5% (F1), 117.3%IA/g, σ = 16.3% (F2), and 167.7%IA/g, σ = 16. 5 (F3), respectively. F3 is significantly higher than F1 and F2 (*p* < 0.001).

**Figure 8:**
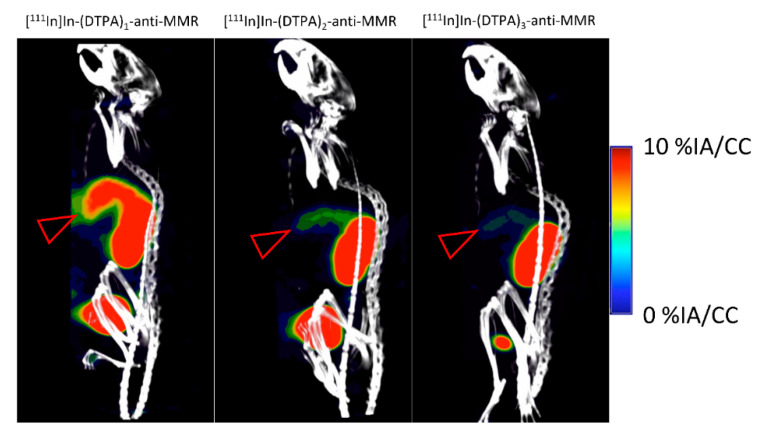
SPECT/CT imaging of [^111^In]In-(DTPA)_n_-anti-MMR fractions at 1 h (p.i.) in 3LLR tumor bearing C57Bl/6 mice provides congruent data as with the ex vivo biodistribution data, where the liver (denoted by red arrow) clearly shows lower signal in fraction 2 and 3 compared to fraction 1. Tumors were not visible at this scaling.

SPECT/CT images were obtained (Figure 8), with the scaling focused on the liver. The images show a clear reduction in signal at the liver site going from fraction 1 to fraction 3, which is likely due to the reduced affinity of fraction 2 and 3. Tumors were not clearly visible at this scaling, while at a lower scaling, tumors were not distinguishable due to an overall body signal, corresponding to uptake of the tracer in several organs.

#### 2.2.5. [^68^Ga]Ga-NOTA-anti-MMR in MMR KO Mice

**Figure 9:**
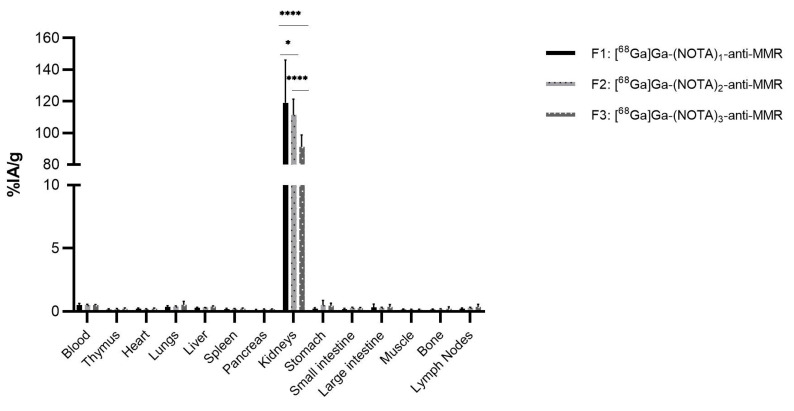
Ex vivo biodistribution analysis of [^68^Ga]Ga-(NOTA)_n_-anti-MMR fractions in C57Bl/6 MMR KO mice (*n* = 4, 5, 4 per group, respectively). (* *p* < 0.05, **** *p* < 0.001).

A biodistribution study of [^68^Ga]Ga-(NOTA)_n_-anti-MMR was performed in naive MMR KO mice to assess the specificity of the tracer and to verify the kidney uptake when no organ uptake occurs (Figure 9). As expected, the overall uptake in organs decreased to background levels, confirming the specificity of each fraction. The kidneys showed uptake values of, respectively, 119.0%IA/g, σ = 27.2% (F1), 111.3%IA/g, σ = 10.0% (F2), and 91.2%IA/g, σ = 7.4% (F3), with F1 being significantly higher compared to F2 (*p* < 0.05) and F3 and F2 being significantly higher than F3 (*p* < 0.001 for both comparisons).

A comparable pattern to the anti-HER2 tracers occurs again for the kidney uptake. This is likely due to the different tracer biodistribution compared to wild-type C57BL/6 mice, which natively express the MMR target. This study in MMR KO mice suggests again that the overall kidney uptake is reduced as the number of chelators increases for the [^68^Ga]Ga-(NOTA)_n_-anti-MMR.

## 3. Discussion

Chemical modifications of proteins in tracer development, such as conjugation to a chelator, can have a profound impact on the physical and pharmacokinetic properties of the tracer. The characterization of such tracers is imperative for clinical usage. While for peptides—as only one compound is obtained at the end of the production process—the characterization of the tracer is relatively simple, this is usually not the case for larger protein-based tracers such as antibody tracers [32]. Lysine-conjugation on sdAbs can also give rise to a heterogenous mixture of sdAbs with a different degree of conjugation [37]. In the design of clinical tracers, random labeling to lysines remains one of the most straightforward techniques. Site-specific conjugation methods have been developed to obtain a homogenous mixture. However, these conjugation methods are usually more complex, require enzymes, such as the sortase A, and/or adaptations of the sdAb sequence, such as addition of linkers, to allow site-specific reactions [38,39,40], resulting in a more challenging translation to GMP manufacturing. For this reason, the sdAbs evaluated in this study have entered clinical trials formulated as a randomly labeled mix of (NOTA)_n_–sdAb conjugates. However, questions may always arise as to the functionality and safety of such a tracer mixture. Indeed, should one of the contained fractions possess a biodistribution profile significantly different from the others, this may present as a problem to regulatory authorities. Therefore, the significance of this study lies in demonstrating that the different fractions contained within one tracer mixture, especially for the clinically relevant NOTA-conjugated sdAbs, overall maintain the expected biodistribution pattern associated with sdAbs. This specifically refers to the rapid renal clearance and fast uptake and accumulation into target-expressing tumors.

In this study, for the first time, the effect of the degree of chelator conjugation on physicochemical properties and in vivo biodistribution of two sdAbs was evaluated after NOTA or DTPA conjugation. By using an AEX method, the separation of different fractions of chelator-conjugated sdAb protein could be achieved, allowing such an assessment. The separation of different fractions is based on interaction with the packing of an AEX column. This type of column has positively charged packing, which allows interaction with the negative charges of the chelators. To break the interaction between the compounds and the packing bed, counter ions (Cl-) are added in a gradient (solvent B). SdAbs with a higher number of chelators will have a stronger interaction with the packing bed and will therefore require more counter ions to detach and consequently a higher gradient of solvent B. As such, a separation of different fractions can be obtained.

On first analysis, differences in several properties were observed for each of these fractions. As could be expected from the conjugation of strongly anionic complexes such as the NOTA and DTPA chelators to the cationic lysine side chain, there was a significant drop in the overall pI of the complexes. Logically, this effect increased with the degree of conjugation. Furthermore, we observed an unequal impact of the conjugation of either chelator on the pI, due to the stronger anionic character of DTPA compared to NOTA. SPR analysis seems to indicate a small impact of the conjugation degree on the affinity, where a higher degree of conjugation led to a decrease in affinity. Of note, for the anti-HER2 sdAb, a crystal structure is available, where it has been confirmed that none of the lysines are part of the paratope [41]. For the anti-MMR sdAb, no crystal structure is available, and so no information is available about lysines being part of the paratope. However, whether the lysines are in the paratope or in the sdAb framework regions, it seems that conjugated chelators can at least to some extent reduce the sdAb binding. Remarkably, a pertinent difference in impact of conjugation on the affinity is observed between the NOTA and DTPA-chelator, for both sdAbs. This could be explained by the size differences between both chelators. The NOTA chelator is smaller compared to CHX-A″-DTPA and has one main rotation point at the nitrogen-ring structure. However, the CHX-A″-DTPA has a more open structure and contains two main rotation points in structure, which makes the CHX-A″-DTPA more flexible and increases the potential space occupancy compared to NOTA. This results in a higher potential hindrance from CHX-A″-DTPA compared to NOTA, which consequently may explain the higher affinity loss.

In vivo biodistribution studies for the anti-HER2 tracers shows a background level signal in most organs and tissue. This is expected, as the anti-HER2 tracers only bind to human HER2 and not the murine HER2. Tumors show a clear uptake of all fractions of both [^68^Ga]Ga-(NOTA)_n_-anti-HER2 and [^111^In]In-(DTPA)_n_-anti-HER2 constructs. However, the selectivity of the anti-HER2 tracer to human HER2 limits a more profound assessment of the impact of conjugation on the affinity. For both [^68^Ga]Ga-(NOTA)_n_-anti-HER2 and the [^111^In]In-(DTPA)_n_-anti-HER2 fractions, a significant difference in kidney uptake was observed, whereby a higher degree of conjugation correlated with a decreased kidney uptake. The kidneys possess a protein reabsorption mechanism to prevent protein loss via the urine [42]. The impact of the conjugation degree on kidney uptake could be caused by altered interaction between the filtered sdAb-conjugates and endocytic receptors in the proximal tubule, which are part of the reabsorption mechanism. These receptors—primarily megalin and cubilin—are known to possess cationic binding motifs [43], which could explain why the more anionic compounds show less uptake. Additionally, the lumen in the proximal tubili is typically rendered negatively charged via active anion secretion [44]. This might repulse more negatively charged compounds (compounds with a lower pI) from the vessel walls and further reduce interaction with reabsorption receptors, leading to a more fluid clearance, than compounds with a higher pI. In line with this hypothesis that the reduced kidney uptake for fraction 2, and more so for fraction 3, is most likely due to a reduced interaction with the re-uptake mechanism in the proximal tubuli, whereby these fractions are more easily flushed out of the kidneys via the urine, no more tracer is available in the rest of the body when reduced kidney accumulation occurs, as can be concluded by the obtained uptake values (especially blood values), which are comparable for all fractions.

The anti-MMR tracers showed uptake in several organs, as these tracers bind to both human and murine MMR. MMR is known to be expressed not only on macrophages but also on endothelial cells [45] and antigen-presenting cells [46] throughout the body, and on Kupffer cells in the liver [47], which results in an uptake in organs such as liver, spleen, pancreas, lungs, lymph nodes, and bone marrow. Previous imaging and biodistribution studies of [^68^Ga]Ga-NOTA-anti-MMR and [^18^F]FB-anti-MMR tracers have shown increased uptake in these organs, while the specificity of the tracer was confirmed in MMR KO mice as the tracer uptake decreased to background or near background levels in all organs [14,24]. The [^68^Ga]Ga-(NOTA)_n_-anti-MMR fractions did not show a significant difference in organ uptake, while significant differences occurred in kidney uptake. However, it seems that F3 consistently shows a lower uptake in organs expressing constitutively MMR compared to F1 and F2, which would correspond with the reduced affinity, measured via SPR. The kidney uptake was the highest for F2, followed by F3 and then F1, with a significant difference between each fraction. The [^111^In]In-DTPA-anti-MMR fractions showed a significant reduction in liver uptake for F2 and F3 compared to F1, which is in concordance with a reduced affinity measured via SPR for F2 and F3. The kidney uptake was the highest for F3, with a significant difference compared to F1 and F2. No difference was obtained between F1 and F2.

The kidney uptake pattern of the anti-MMR tracers in C57BL/6 mice was highly different than that of the anti-HER2 tracers (athymic nude mice) and did not correspond with the trend that a higher conjugation degree leads to a lower kidney uptake. We hypothesized that the difference in mass distribution of the anti-MMR tracers influenced the kidney uptake. Due to variations in organ uptake between the fractions, different amounts are filtered in the kidneys in the same period. For example, F3 of DTPA-anti-MMR showed a consistently lower organ uptake, leading to a higher amount being filtered through the kidneys compared to F1 and F2, which could cause the observed increased kidney uptake. To verify this hypothesis and to confirm the specificity of the tracer, an additional in vivo study of the [^68^Ga]Ga-(NOTA)_n_-anti-MMR fractions was performed in C57Bl/6 MMR KO mice. In this study, the organ uptake was reduced to background levels, confirming the specificity of each fraction, while for the kidney uptake, the same pattern re-emerged as for the anti-HER2 tracers, where a higher degree of conjugation leads to reduced kidney uptake. This seems to confirm our hypothesis that reduced affinity leads to a reduced uptake in different MMR expressing organs and so to more unbound tracer, which will be filtered by the kidneys, leading to higher kidney uptake.

To further investigate the observed differences in kidney uptake, an additional in vivo study could be performed comparing the kidney uptake of (chelator)_n_-sdAb fractions where the chelators have been saturated with the cold isotope after radiolabeling. Complexation of the chelator with a positive metal ion neutralizes (partially) the negative charges. During radiolabeling, the amount of radionuclide is typically far inferior to the number of available chelators, leaving the majority of chelators uncomplexed and negatively charged. However, after saturation, and coinciding neutralization of the chelator’s negative charges, the kidney uptake should increase according to previous explanation about altered interaction with the reabsorption mechanism described above, at least for (chelator)_2_-sdAb and (chelator)_3_-sdAb fractions, provided that the ‘free’ chelators do not saturate in vivo by endogenous metals, such as iron or zinc.

Despite the observed differences between fractions (mainly on kidney uptake), in this study, we could not detect a significant impact of the conjugation degree on tumor targeting of any evaluated tracer. While this is supported by what is conventionally expected for small ligands, where an ideal affinity range is defined as 1–10 nM for optimal tumor uptake and intratumoral distribution, it is also known that a decreased pI can cause diminished tumor uptake, at least in the case of antibodies [30,31]. However, this effect was not observed here for the sdAbs. To assess more in-depth the impact of the conjugation degree on the in vivo targeting and on pharmacokinetics, follow-up studies could be performed, where the sample size determination is focused on measuring differences in target organs, such as the tumor, and where the biodistribution is evaluated at different timepoints.

To summarize, the ability to separate and investigate the different conjugated fractions of an sdAb can be valuable to analyze whether the biodistribution or targeting is affected. In this study, we show that for both the anti-HER2 and anti-MMR sdAb, up to 3 NOTA-chelators can be conjugated to one sdAb without majorly affecting the biodistribution and impeding their usage. The current clinical manufacturing protocol of the NOTA-sdAbs yields a mixture, respectively for the anti-HER2 sdAb [37], of (NOTA)_0_-, (NOTA)_1_-, (NOTA)_2_-anti-HER2, with a low amount of (NOTA)_3_-anti-HER2, and for the anti-MMR sdAb [24] of (NOTA)_0_- and (NOTA)_1_-anti-MMR. The obtained results on the different fractions in this study support the further use of these mixtures in clinical setting. For future sdAbs, the AEX separation method can be applied in preclinical development to characterize fractions so that conjugation conditions can be adjusted if necessary, to obtain the desired mixture of fractions. Importantly, depending on the native presence of the antigen and on the influence of the conjugation on the affinity, the kidney uptake can vary, due to a different mass distribution, as was the case for the anti-MMR constructs. A factor to take into account would be the target-to-kidney ratio, which is ideally as high as possible. Particularly in a therapeutic setting, the decreased kidney uptake associated with a higher degree of conjugation can be valuable to reduce the absorbed dose of the kidneys, which is often the dose-limiting organ for sdAb tracers [41,48,49], as long as the target-to-kidney ratio and therapeutic index remain optimal. Finally, the separation method described here could also be applied as purification method to obtain a single fraction as a final product with the goal to obtain a more homogeneous product. For example, this could be valuable for sdAbs that are more affected by random conjugation than those evaluated in this study.

## 4. Materials and Methods

All commercially obtained chemicals were of analytical grade. Recombinant sdAb-proteins were produced without terminal tags by the Vlaams Instituut voor Biotechnologie (VIB) Protein Service Facility (VIB, Gent) in Pichia pastoris and were formulated in phosphate-buffered saline 1× (PBS) (0.01 M phosphate buffer/0.14 M NaCl) pH 7.4 during the final batch purification. p-SCN-Bn-NOTA and p-SCN-Bn-CHX-A″-DTPA were purchased from Macrocyclics (Macrocyclics, Inc., Plano, TX, USA). ^68^Ga was obtained from a ^68^Ge/^68^Ga Galli EoTM generator (IRE Elit, Fleurus, Belgium), indium chloride ([^111^In]InCl_3_) from Curium (Hertogenbosch, The Netherlands). High-purity water for trace analysis (TraceSELECT, Riedel-de-Haën, Honeywell Research Chemicals, Seelze, Germany) was used for the preparation of radiolabeling buffer.

### 4.1. NOTA/DTPA-Conjugation

SdAbs (‘2Rs15d’ anti-HER2 sdAb: 10 mg, 0.79 μmol; ‘MMR3.49’ anti-MMR sdAb: 10 mg, 0.79 μmol) were buffer-exchanged to 0.5 M sodium carbonate/0.15 M sodium chloride buffer (sodium carbonate anhydrous—sodium hydrogen carbonate—sodium chloride, VWR Chemicals, Leuven, Belgium), pH 8.7 (NOTA-conjugation), or pH 9.5 (DTPA-conjugation), using PD-10 size exclusion disposable columns (GE Healthcare, Buckinghamshire, UK). A thirty-fold molar excess of p-SCN-Bn-NOTA or p-SCN-Bn-CHX-A″-DTPA was added to the protein solution (2.2–2.4 mg/mL) and incubated for 2–2.5 h at room temperature (RT).

#### 4.1.1. SEC Purification

Size exclusion chromatography (SEC) was performed using a Superdex Peptide 10/300GL (GE Healthcare Bio-Sciences AB, Uppsala, Sweden) column with 0.02 M Tris (Sigma-Aldrich, St. Louis, MO, USA) pH 7.5 as mobile phase (0.5 mL/min) for purification of the conjugated sdAb from excess chelator (detection of UV absorption at 280 nm). NOTA- or DTPA-conjugated sdAbs were injected, and peaks corresponding to monomeric functionalized NOTA- or DTPA-sdAb were collected and concentration determined by UV spectrometry. SEC was also used for quality control of the conjugated sdAb.

#### 4.1.2. AEX Purification

Anion exchange chromatography (AEX) was performed using an ENrich Q 5 × 50 column (Bio-Rad Laboratories, Inc., California, CA, USA) with 0.02 M Tris (≥99.8%, Sigma-Aldrich Chemie, Steinhelm, Germany) adjusted to pH 7.5 with 0.1 M HCl (Hydrochloric acid, ≥37% puriss. p.a., Ph.Eur., Sigma-Aldrich Chemie, Steinhelm, Germany) as solvent A and 0.25 M or 0.4 M NaCl as solvent B for NOTA-sdAb and DTPA-sdAb, respectively, (1.5 mL/min). For NOTA-anti-HER2, DTPA-anti-HER2, and DTPA-anti-MMR compounds, a gradient method was used starting with 100% solvent A and 0% solvent B, gradually increasing solvent B up to 100% over 60 mL. For the NOTA-anti-MMR compound, solvent B was gradually increased up to 60% over 60 mL, which was followed by 3 mL of 100% solvent B. Samples of approximately 3 mg of NOTA- or DTPA-conjugated sdAb (after SEC purification) were injected, and peaks absorbing over 75 mAU (detection at 280 nm) were collected.

The collected peaks corresponding to either (chelator)1-sdAb, (chelator)2-sdAb, or (chelator)3-sdAb were pooled accordingly, providing fraction 1, fraction 2, and fraction 3, respectively. Each fraction was further buffer exchanged to pure water using a Vivaspin 2 concentrator (Sartorius Stedim Lab, Stonehouse, UK) with a molecular weight cut-off of 5 kDa; then, their concentration was determined by measuring the absorption at 280 nm using a Nanodrop One (Thermo Fisher Scientific). Parameters for each fraction are available in supplemental data (Supplemental Table S1).

### 4.2. Mass Spectrometry Analysis

The degree of NOTA or DTPA conjugation to the sdAb was determined by ESI-Q-ToF-MS at the GIGA Proteomics Facility at the University of Liège. Briefly, samples were ultrafiltrated with a 3 kDa cut-off membrane to remove salts and then re-diluted in a 30% ACN, 0.5% formic acid, 25 mM ammonium acetate solution. The analysis was performed on a Synapt G2 HDMS mass spectrometer (Waters) in positive ion mode.

### 4.3. Surface Plasmon Resonance Analysis

Surface Plasmon Resonance (SPR) affinity determination was performed on a Biacore T200 (GE Healthcare Bio-Sciences AB, Uppsala, Sweden) system as described previously [14,36]. Briefly, a CM5 chip was coated with either recombinant HER2-Fc (Sino biological, Beijing, China) or recombinant hMMR (RnDSystems, Minneapolis, MN, USA) protein via 1-ethyl-3-(-3-dimethylaminopropyl)carbodiimide (EDC) and N-hydroxysuccinimide (NHS) chemistry. The affinity was determined by flowing different concentrations of precursor over the immobilized protein. The obtained curves were fitted with a 1:1 sdAb:antigen binding model to calculate the binding parameters. A reference sample containing unconjugated anti-HER2-His_6_ or anti-MMR-His_6_, stored at −20 °C, was added during each run.

### 4.4. Iso-Electric Focusing Electrophoresis

Iso-electric focusing (IEF) electrophoresis was performed using a vertical precast gel with pH range from 3 to 10 (SERVAGel IEF 3–10, SERVA Electrophoresis GmbH, Heidelberg, Germany). Samples were prepared according to the manufacturer’s protocol. Briefly, 2 µg of conjugated sdAb was mixed at least 1:1 with IEF sample buffer (SERVA Electrophoresis GmbH, Heidelberg, Germany) to a max volume of 35 µL, after which the samples were loaded in the gel. The inner chamber of the electrophoresis chamber was filled with IEF cathode buffer (SERVA Electrophoresis GmbH, Heidelberg, Germany), while the outer chamber was filled with IEF anode buffer (SERVA Electrophoresis GmbH, Heidelberg, Germany). Gels were run using following program: 1 h at 100 V, 2 h at 200 V, and finally, 30 min at 500 V. Afterwards, the gel was fixated in a 20% tricholoracetic acid (Acros Organics, Geel, Belgium) solution for 20 min, followed by staining in a crystal violet (SERVA Violet 17, SERVA Electrophoresis GmbH, Heidelberg, Germany)/20% phosphoric acid (Acros Organics, part of Thermo Fisher Scientific, Geel, Belgium) solution for 10 min. Then, the gel was destained in 3% phosphoric acid solution until a clear background was obtained. The gels were imaged using an Amersham 680RGB imager (GE Healthcare Bio-Sciences AB, Uppsala, Sweden) and analyzed via the GE ImageQuant TL 1D v 8.2.0 analysis software.

### 4.5. Radiolabeling

For the ^68^Ga labeling, per fraction, 30 µg (NOTA)_n_-sdAb (*n* = 1 or 2 or 3) was diluted in 250 µL 1 M NaOAc (sodium acetate trihydrate, ≥99.5%, puriss. p.a., Ph.Eur., Sigma-Aldrich Chemie, Steinhelm, Germany—acetic acid, ≥99.8%, puriss. p.a., Ph.Eur., Sigma-Aldrich Chemie, Steinhelm, Germany), pH 5.0, and 250 µL of [^68^Ga]GaCl_3_ eluate was added to the precursor. The solution was incubated at RT for 10 min. The radiochemical purity (RCP) was assessed on binderless glass microfiber paper that was impregnated with silica gel (instant thin layer chromatography, iTLC-SG) (Agilent Technologies, Diegem, Belgium) with 0.1 M sodium citrate pH 5 (citric acid, trisodium salt, dihydrate—citric acid, monohydrate, Acros Organics, part of Thermo Fisher Scientific, Geel, Belgium) as mobile phase.

Afterwards, the solution was purified via NAP-5 (GE Healthcare, Buckinghamshire, UK) and eluted in 1 mL PBS (0.01 M phosphate buffer/0.14 M NaCl) (PBS Tablets, Merck, Darmstadt, Germany). The final solution was filtered through 0.22 µm Millex filter (Merck Millipore Ltd., Carrigtwohill, Ireland) before preparation of the syringes.

For the ^111^In-labeling, per fraction, 50 µg (DTPA)_n_-sdAb (*n* = 1 or 2 or 3) was diluted in 250 µL 0.2 M NH_4_OAc (≥99.99% trace metal basis, Honeywell, Seelze, Germany), adjusted to pH 5.0 with 0.1 M HCl, and 250 µL [^111^In]InCl_3_ was added to the precursor. The solution was incubated at 37 °C for 30 min. iTLC was performed to assess the RCP. Afterwards, the solution is purified via NAP-5 and collected in 1 mL PBS (0.01 M phosphate buffer/0.14 M NaCl). The final solution is filtered through 0.22 µm Millex filter.

### 4.6. Cell Culturing

The human ovarian cancer cell line SKOV3 (HER2+) was obtained from American Type Culture Collection (ATCC, Manassas, VA, USA), and the cells were cultured as described previously [50]. The murine lung adenocarcinoma cell line 3LL-R, a subclone of the Lewis Lung carcinoma (LLC) cell line [51], a kind gift from Prof. Damya Laoui from the lab of Cellular and Molecular Immunology (CMIM, Vrije Universiteit Brussel, Brussels, Belgium), was cultured in RPMI containing Pen/Strep (1%), L-Glu (1%), non-essential amino acids (1%), and 10% FBS (Gibco, Life Technologies, Paisly, UK).

### 4.7. In Vivo Biodistribution Study

The mice were housed at 22 °C in 50–60% humidity with a light/dark cycle of 12 h in individually ventilated cages. Food pellets and water were provided ad libitum.

For the tumor-targeting study of anti-HER2 constructs, 6-week-old, athymic, immune-deficient, female Crl:NU(NCr)-Foxn1^nu^ mice (Charles River, Écully, France) were inoculated subcutaneously at the right flank with 10^7^ HER2-expressing SKOV-3 cells, and tumors were grown for 6–8 weeks with tumor volumes ranging between 50 and 250 mm³. For the tumor-targeting study of anti-MMR constructs, 6-week-old, healthy, female C57BL/6J mice (Charles River, Écully, France) were inoculated subcutaneously at the right flank with 10^6^ 3LL-R clone of LLC cells. Tumors were grown for 11 days with tumor volumes ranging between 150 and 250 mm³. For each construct, mice were equally and randomly distributed over three groups with *n* = 6 per fraction. For the NOTA-anti-MMR construct, the mice were further divided into two separate cohorts (*n* = 3 per fraction per cohort). Unfortunately, due to experimental error, the first cohort of mice was injected with impure fractions. These mice were omitted from analysis, resulting in only *n* = 3 per fraction in the final analysis for this construct.

For the specificity study of the NOTA-anti-MMR compounds, 4–7-month-old female C57BL/6 MMR knock-out (KO) mice were used, kindly provided by Prof. Damya Laoui from the CMIM lab (Vrije Universiteit Brussel, Brussels, Belgium). Thirteen mice were of suitable age for the study, while the breeding was being discontinued. The MMR KO mice were randomly divided into three groups with *n* = 4, 5, and 4 for fraction 1 (F1), fraction 2 (F2), and fraction 3 (F3), respectively.

All mice were injected with ± 5 µg (0.37 nmol, σ = 0.01) of radiolabeled tracer (5–20 MBq) (Supplemental Tables S2 and S3). Mice injected with an ^111^In-labeled compound were subjected to SPECT/CT imaging 1 h post injection (p.i.). All mice were sacrificed via neck dislocation 80 min p.i. for organ collection. The organs were weighed and measured for radioactivity in a gamma-counter (Cobra II, Packard for anti-HER2 studies and Wizard2 Gamma Counter, Perkin Elmer for anti-MMR studies). Tissue/organ uptake was calculated and expressed as percentage injected activity per gram (%IA/g), corrected for decay. A%IA/g ≤ 0.5% is considered as background signal. All handlings on living animals were performed while the animals were under anesthesia by isoflurane (ABBOTT, Ottignies-LLN Belgium) (5% induction in a box and 2.5% maintenance via a nose cone).

### 4.8. SPECT/CT

Mice were imaged 1 h p.i. of the tracer using a Vector+ SPECT/CT system (MIlabs, Utrecht, The Netherlands) equipped with a general purpose rat/mouse 1.5 mm 75 pinhole collimator. SPECT imaging was performed in spiral mode with 6 bed positions and an acquisition time of 200 s per bed position. Images were reconstructed with 2 subsets and 4 iterations with a voxel size of 0.4 mm in MIlabs reconstruction software v. 8.00. A CT scan was performed in 1 bed position with a duration of 146 s at 60 kV and a pixel size of 80 µm. Reconstructed images were further processed via AMIDE software v1.0.5 (SourceForge), while 3D images were made using OsirixMD software v11.0.0 (Pixmeo, Bernex, Switzerland).

### 4.9. Statistical Analysis

A power analysis was performed to determine the sample size, with a focus on measuring differences in kidney uptake, assuming a normal distribution. The results yielded a sample size of 6 animals per group.

For statistical analysis on the ex vivo biodistribution data, a two-way ANOVA statistical test with multiple comparisons was performed using Graphpad Prism v8.3.1 (Graphpad Software, LLC, San Diego, CA, USA).

## 5. Conclusions

Anion exchange chromatography has shown to be a viable method for the separation of sdAb-based tracers with different degrees of chelator conjugation. This allows further characterization of a tracer and optimization of the conjugation conditions to obtain a desired final mixture. Here, we evaluated two sdAbs that are currently undergoing clinical translation, and they have demonstrated that while the affinity is mildly impacted by increasing degrees of conjugation, it remains within the low nanomolar range, with minimal effect on tumor targeting and overall biodistribution profile. Interestingly, a higher degree of conjugation also reduces the degree of renal uptake, which can only be considered positive in the context of radiopharmaceuticals. These findings indicate that random conjugation can be an appropriate design strategy for clinical sdAb tracers.

## Figures and Tables

**Table 1:** Affinity measurement by SPR. Reference corresponds to the unconjugated sdAb; fractions 1, 2, and 3 refer to sdAb with 1, 2, and 3 conjugated chelators, respectively.

Compound	Affinity—K_D_ (nM)				
	Reference *	Mixture **	Fraction 1	Fraction 2	Fraction 3
NOTA-anti-HER2	3.7, σ = 0.7	4.8, σ = 1.6	4.1	5.0	7.4
DTPA-anti-HER2	3.7, σ = 0.7	NA ***	3.3	6.2	10.0
NOTA-anti-MMR	1.2, σ = 0.3	1.2, σ = 0.2	1.3	1.7	2.9
DTPA-anti-MMR	1.2, σ = 0.3	NA ***	1.6	2.4	4.8

* Reference compound is the respective unconjugated anti-HER2 or anti-MMR sdAb, the value is based on historical data. ** SPR analysis of the typical mixture obtained upon conjugation following the current clinical protocol [24,37] the value is based on historical data. *** Not available.

**Table 2:** Determination of isoelectric point by IEF gel.

Compound	pI			
	Reference *	Fraction 1	Fraction 2	Fraction 3
NOTA-anti-HER2	>8.3	5.2, 5.0	5.0, 4.1, 3.5	3.5, 3.4
DTPA-anti-HER2	>8.3	5.0, 4.5	3.5, 3.3	3.1, 2.9
NOTA-anti-MMR	>8.3	5.4, 5.2, 4.9	4.7, 3.7, 3.5	3.5, 3.2
DTPA-anti-MMR	>8.3	5.0, 4.8, 3.8	3.4, 3.1	3.1, 2.8

* Reference compound is the respective unconjugated anti-HER2 or anti-MMR sdAb.

## Data Availability

The raw data and processed data required to reproduce these findings are available to download from https://vub.sharepoint.com/:f:/r/teams/ORG-ICMI/External%20Share/Shared%20Documents/2021_Baudhuin%20et%20al_IEX?csf=1&web=1&e=jn3dAT (access on 23 April 2021). Access can be granted upon request.

## Supplementary Materials

The following are available online at https://www.mdpi.com/article/10.3390/ph14050407/s1. Table S1: Parameters for concentration measurement via UV spectrophotometry; Table S2: Number of moles of compound use for radiolabeling and injection; Table S3: Injected activities of each fraction per mouse; Table S4: Setup Iso-electric focusing gel electrophoresis; Table S5: Ex vivo biodistribution data [68Ga]Ga-NOTA-anti-HER2; Table S6: Ex vivo biodistribution data [111In]In-DTPA-anti-HER2; Table S7: Ex vivo biodistribution data [68Ga]Ga-NOTA-anti-MMR; Table S8: Ex vivo biodistribution data [111In]In-DTPA-anti-MMR; Table S9: Ex vivo biodistribution data [68Ga]Ga-NOTA-anti-MMR in KO mice. Figure S1A. Mass spectrometry spectrum of (NOTA)1-anti-HER2 (theoretical mass: 13,078); Figure S1B. Mass spectrometry spectrum of (NOTA)2-anti-HER2 (theoretical mass: 13,528); Figure S1C. Mass spectrometry spectrum of (NOTA)3-anti-HER2 (theoretical mass: 13,978); Figure S2A. Mass spectrometry spectrum of (DTPA)1-anti-HER2 (theoretical mass: 13,228); Figure S2B. Mass spectrometry spectrum of (DTPA)2-anti-HER2 (theoretical mass: 13,828); Figure S2C. Mass spectrometry spectrum of (DTPA)3-anti-HER2 (theoretical mass: 14,428); Figure S3A. Mass spectrometry spectrum of (NOTA)1-anti-MMR (theoretical mass: 13,128); Figure S3B. Mass spectrometry spectrum of (NOTA)2-anti-MMR (theoretical mass: 13,578); Figure S3C. Mass spectrometry spectrum of (NOTA)3-anti-MMR (theoretical mass: 14,028); Figure S4A. Mass spectrometry spectrum of (DTPA)1-anti-MMR (theoretical mass: 13,278); Figure S4B. Mass spectrometry spectrum of (DTPA)2-anti-MMR (theoretical mass: 13,878); Figure S4C. Mass spectrometry spectrum of (DTPA)3-anti-MMR (theoretical mass: 14,478); Figure S5. Imaging Iso-electric focusing gel electrophoresis.
