# Supplementary material for: Decorating sdAbs with Chelators: Effect of Conjugation on Biodistribution and Functionality
Source: Pharmaceuticals (Basel). 2021 Apr 25;14(5):407. doi: 10.3390/ph14050407 (PMC8146233; doi:10.3390/ph14050407)
Supplement: Supplementary file 1 [file pharmaceuticals-14-00407-s001.zip › pharmaceuticals-1202185-supplementary.pdf]

pharmaceuticals

Article

Decorating sdAbs with Chelators: Effect of Conjugation on Biodistribution and Functionality

Henri Baudhuin [1,*], Janik Puttemans [1], Heleen Hanssens [1], Philippe Vanwolleghem [1], Sophie Hernot [1], Geert Raes [2,3], Catarina Xavier [1], Tony Lahoutte [1,4] and Pieterjan Debie [1]

1 Department of Medical Imaging (MIMA), Vrije Universiteit Brussel, Laarbeeklaan 103, B-1090 Brussels, Belgium; Janik.Puttemans@vub.be (J.P.); Heleen.Hanssens@vub.be (H.H.); philippe.vanwolleghem@ppms.be (P.V.); Sophie.Hernot@vub.be (S.H.); Catarina.Xavier@vub.be (C.X.); Tony.Lahoutte@uzbrussel.be (T.L.); Pieterjan.Debie@vub.be (P.D.)
2 Unit of Cellular and Molecular Immunology (CMIM), Vrije Universiteit Brussel, Pleinlaan 2, B-1050 Brussels, Belgium; Geert.Raes@vub.be
3 Myeloid Cell Immunology Laboratory, VIB Center for Inflammation Research, Pleinlaan 2, B-1050 Brussels, Belgium
4 Nuclear Medicine Department (NUCG), Universitair Ziekenhuis Brussel (UZ Brussel), Laarbeeklaan 101, B-1090 Brussels, Belgium
* Correspondence: Henri.Baudhuin@vub.be; Tel.: +32-2-477-49-91

Supplemental data

Table S1. Parameters for concentration measurement *via* UV spectrophotometry.

Compound	Chelator	# of Chelator/sdAb	Extinction coefficient ($M^{-1}cm^{-1}$) at 280 nm	Molecular weight (kDa)
Anti-HER2	NOTA	1	41.44	13.08
		2	57.44	13.53
		3	73.44	13.98
	DTPA	1	42.44	13.23
		2	59.44	13.83
		3	76.44	14.43
Anti-MMR	NOTA	1	40.66	13.13
		2	56.66	13.58
		3	72.66	14.03
	DTPA	1	41.66	13.28
		2	58.66	13.88
		3	75.66	14.48

Table S2. Number of moles of compound use for radiolabeling and injection.

Compound	Chelator	# of Chelator/sdAb	Moles used for radiolabeling (nmol)	Moles injected per mouse (nmol)
Anti-HER2	NOTA	1	2.29	0.38
		2	2.22	0.37
		3	2.15	0.36
	DTPA	1	3.78	0.38
		2	3.62	0.36
		3	3.47	0.35
Anti-MMR	NOTA	1	2.28	0.38
		2	2.21	0.37
		3	2.14	0.36
	DTPA	1	3.77	0.38
		2	3.60	0.36
		3	3.45	0.35

Table S3. Injected activities of each fraction per mouse.

Compound	N/group	1 chelator-fraction	2 chelator-fraction	3 chelator-fraction
[^{68}Ga]Ga-(NOTA)$_n$-anti-HER2-sdAb	3	12.6 MBq, σ = 0.7 MBq	12.4 MBq, σ = 0.4 MBq	11.6 MBq, σ = 0.4 MBq
[^{111}In]In-(DTPA)$_n$-anti-HER2-sdAb	6	9.3 MBq, σ = 0.4 MBq	9.4 MBq, σ = 0.6 MBq	10.0 MBq, σ = 0.7 MBq
[^{68}Ga]Ga-(NOTA)$_n$-anti-MMR-sdAb	6	17.3 MBq, σ = 0.1 MBq	13.5 MBq, σ = 0.2 MBq	11.7 MBq, σ = 0.3 MBq
[^{111}In]In-(DTPA)$_n$-anti-MMR-sdAb	6	9.7 MBq, σ = 0.3 Mbq	10.1 MBq, σ = 0.3 MBq	8.9 MBq, σ = 0.3 MBq
[^{68}Ga]Ga-(NOTA)$_n$-anti-MMR-sdAb KO mice	4/5/4	9.0 MBq, σ =0.3 MBq	7.2 MBq, σ = 0.2 MBq	5.9 Mbq, σ = 0.2 Mbq

Mass spectrometry data

1. NOTA-anti-HER2

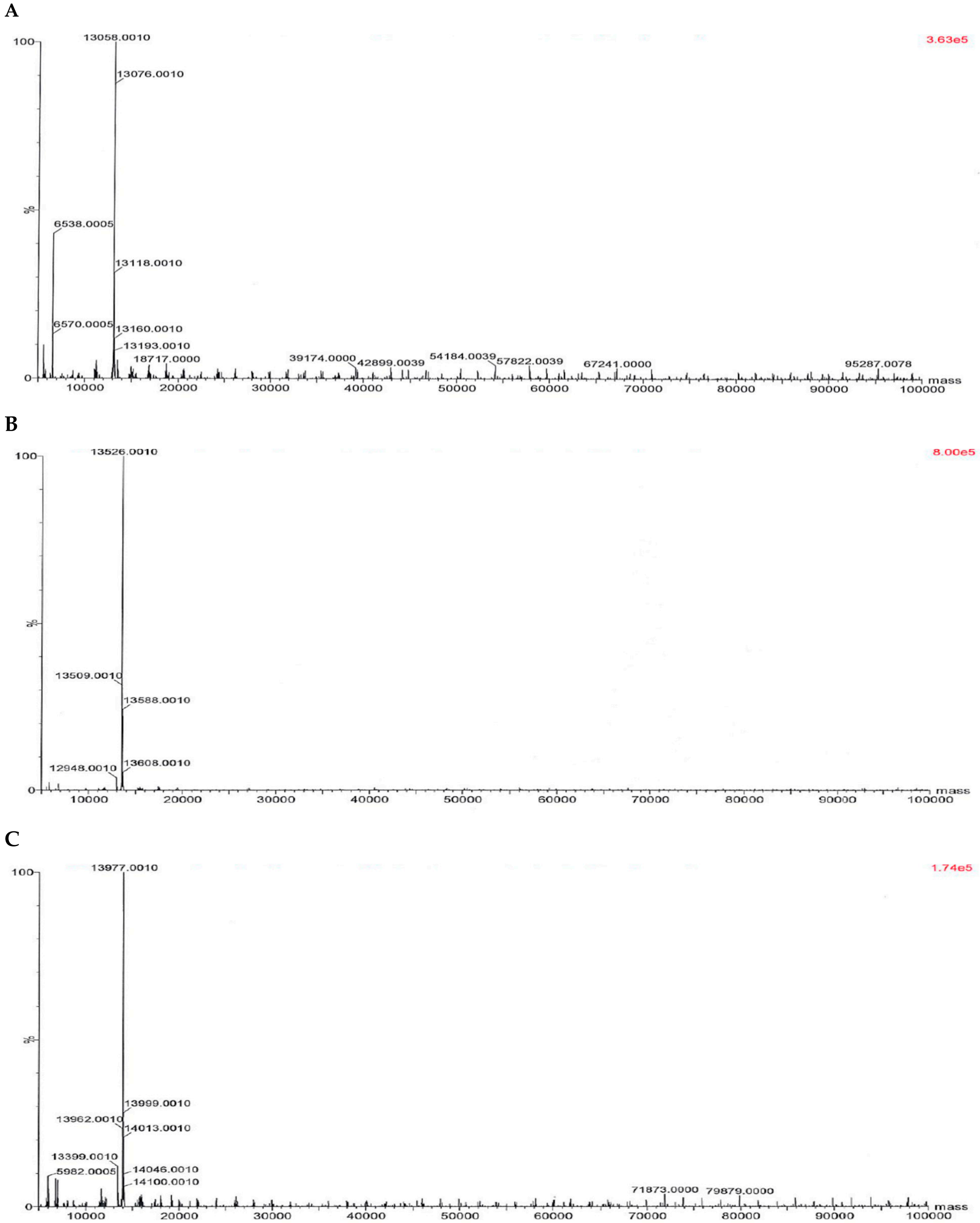

Figure S1. Mass spectrometry spectrum of (A) $(NOTA)_1$-anti-HER2 (theoretical mass: 13,078), (B) $(NOTA)_2$-anti-HER2 (theoretical mass: 13,528) and (C) $(NOTA)_3$-anti-HER2 (theoretical mass: 13,978).

2. DTPA-anti-HER2

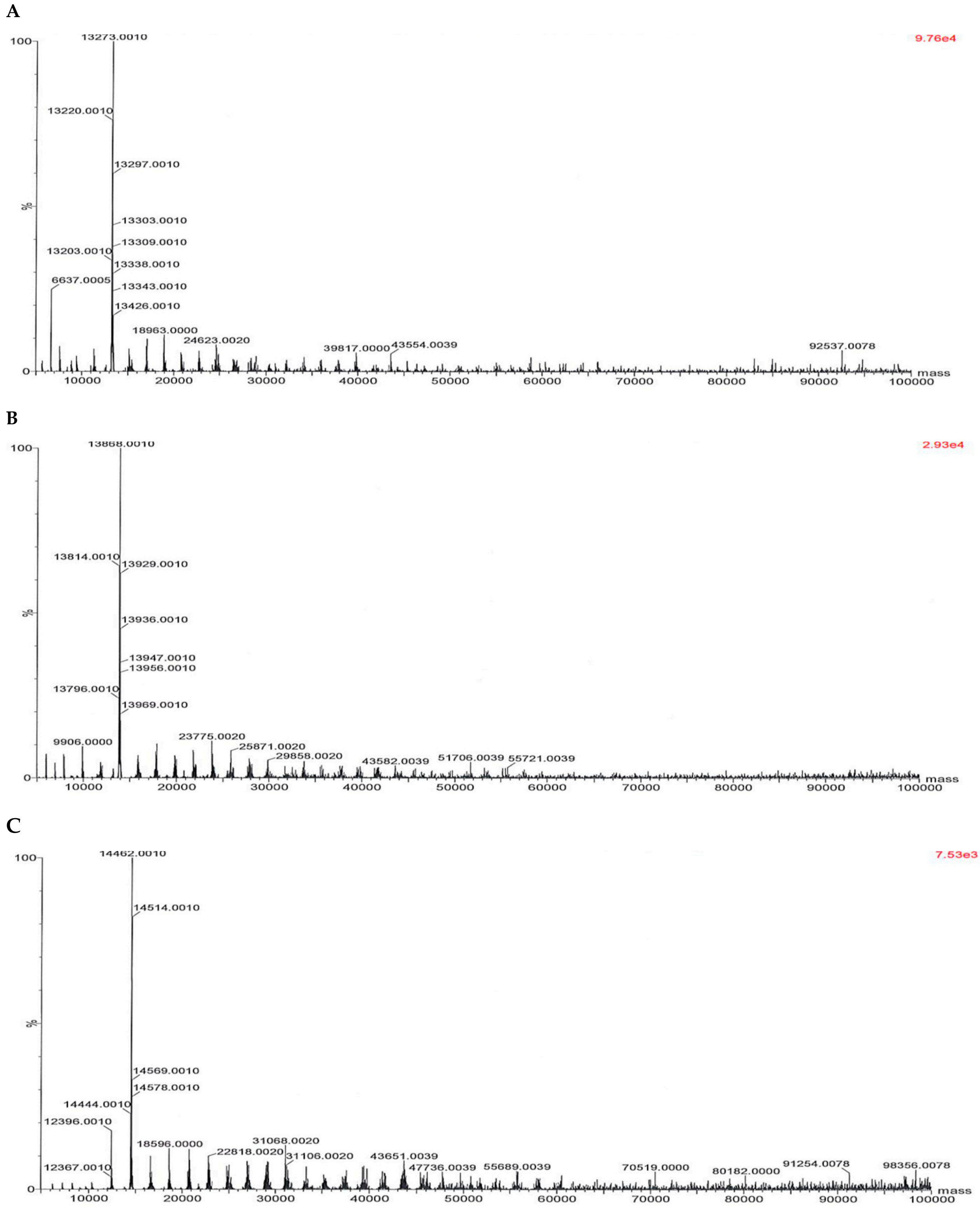

Figure S2. Mass spectrometry spectrum of (A) (DTPA)1-anti-HER2 (theoretical mass: 13,228), (B) (DTPA)2-anti-HER2 (theoretical mass: 13,828) and (C) (DTPA)3-anti-HER2 (theoretical mass: 14,428).

3. <u>NOTA-anti-MMR</u>

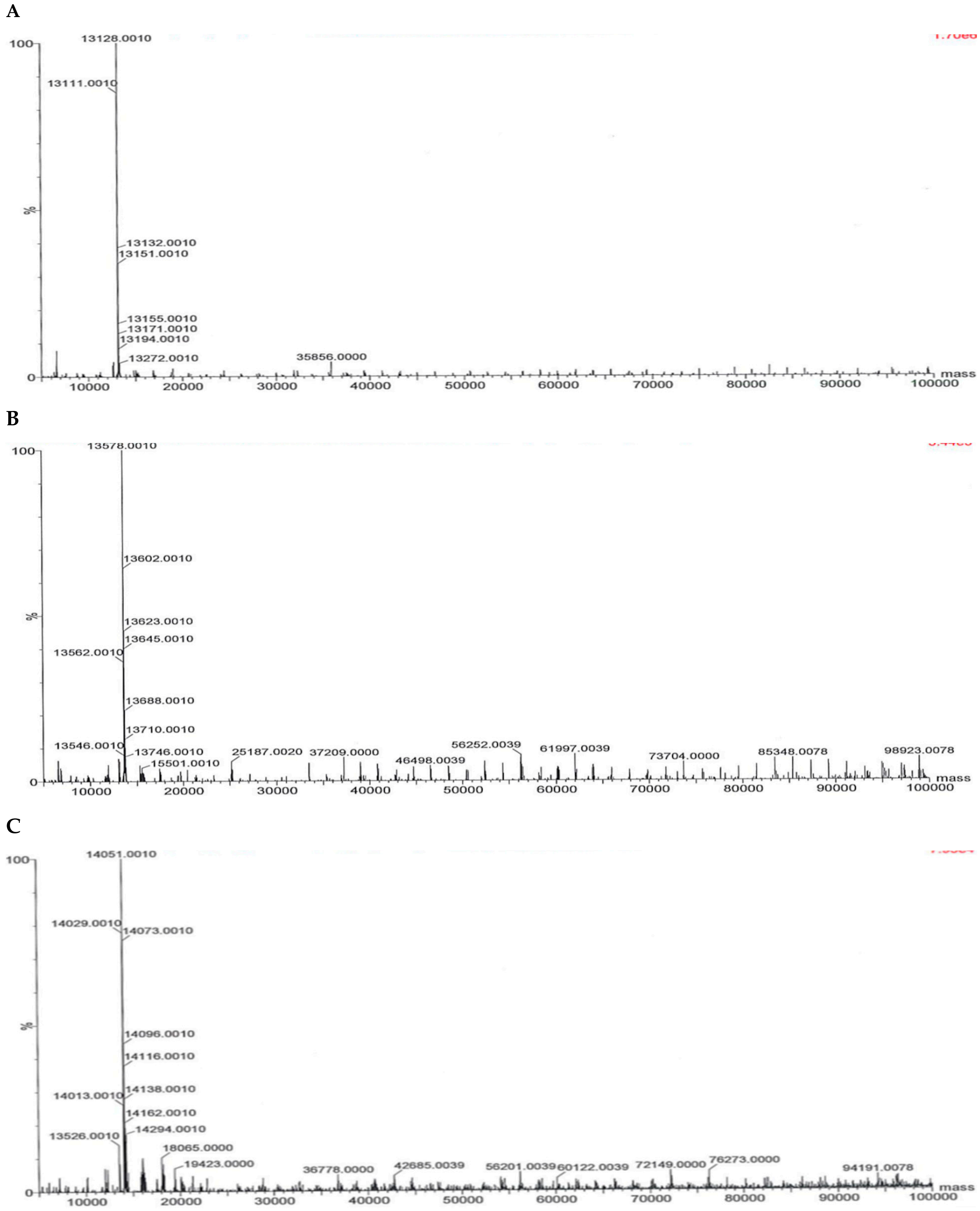

Figure S3. Mass spectrometry spectrum of (A) $(NOTA)_1$-anti-MMR (theoretical mass: 13,128), (B) $(NOTA)_2$-anti-MMR (theoretical mass: 13,578) and (C) $(NOTA)_3$-anti-MMR (theoretical mass: 14,028).

4. <u>DTPA-anti-MMR</u>

A

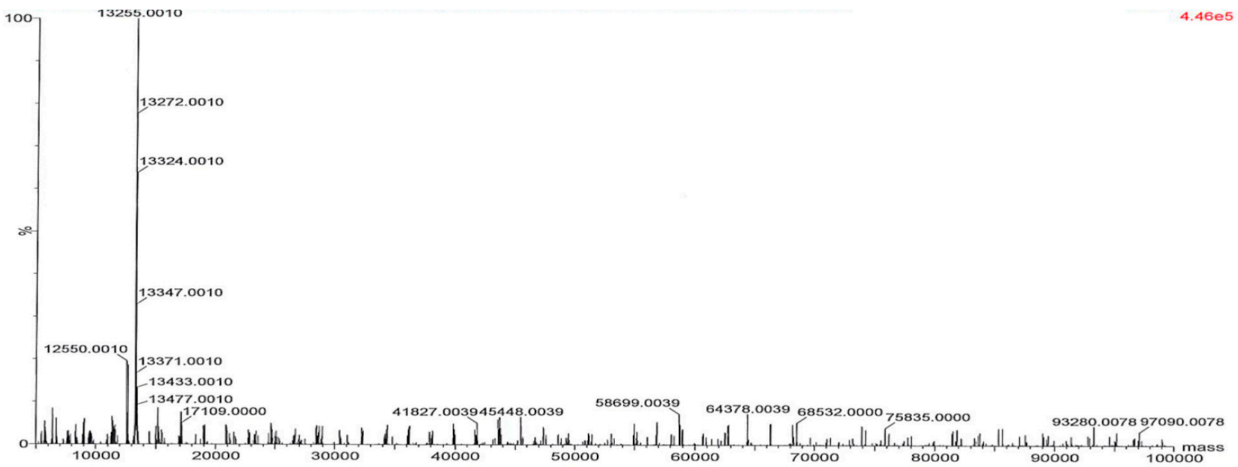

B

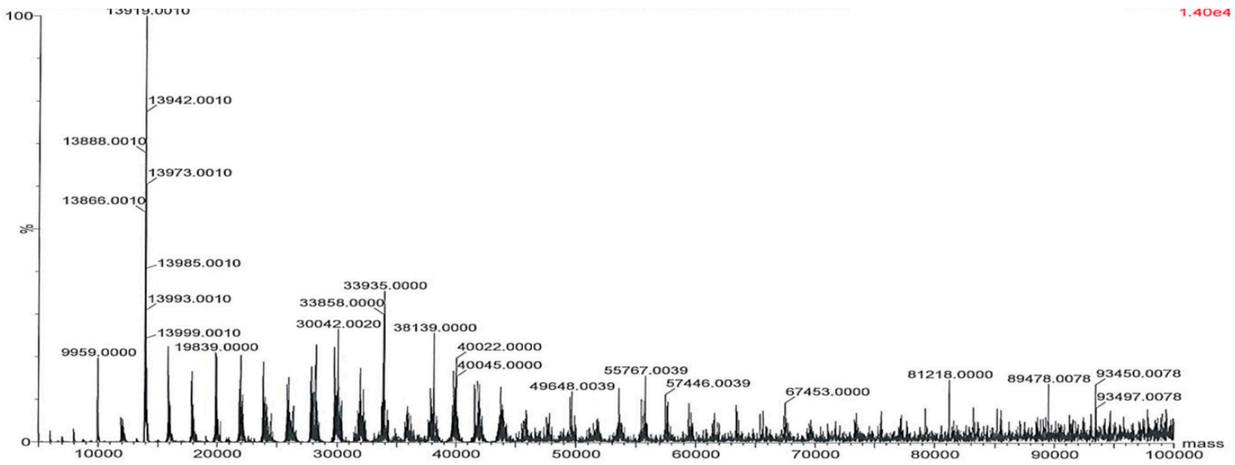

<u>C</u>

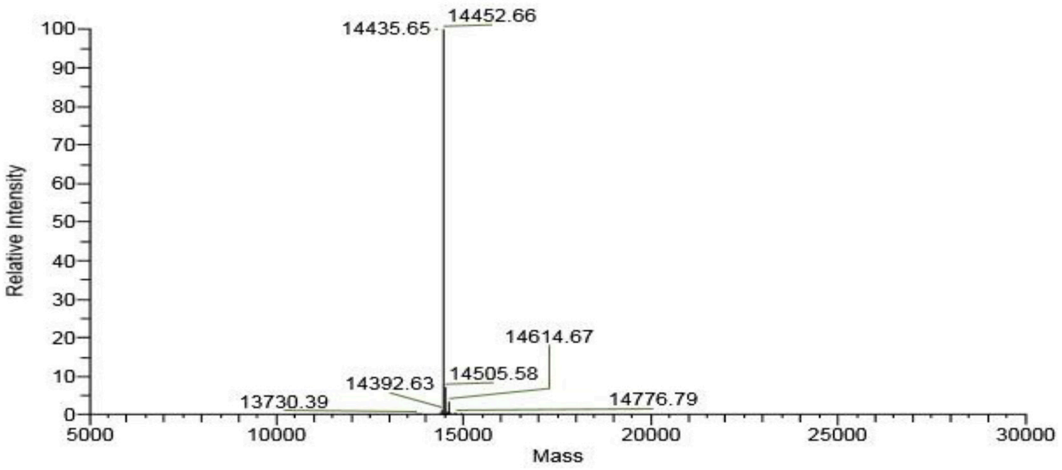

Figure S4. Mass spectrometry spectrum of (A) $(DTPA)_1$-anti-MMR (theoretical mass: 13,278), (B) $(DTPA)_2$-anti-MMR (theoretical mass: 13,878) and (C) $(DTPA)_3$-anti-MMR (theoretical mass: 14,478).

Isoelectric focusing

Table S4. Setup Iso-electric focusing gel electrophoresis.

Lane	1	2	3	4	5
Setup Gel A	Ladder	$(NOTA)_1$-anti-HER2	$(NOTA)_2$-anti- HER2	$(NOTA)_3$-anti- HER2	Ladder
Setup Gel B	Ladder	$(DTPA)_1$-anti- HER2	$(DTPA)_2$-anti- HER2	$(DTPA)_3$-anti- HER2	Ladder
Setup Gel C	Ladder	$(NOTA)_1$-anti-MMR	$(NOTA)_2$-anti-MMR	$(NOTA)_3$-anti-MMR	Ladder
Setup Gel D	Ladder	$(DTPA)_1$-anti-MMR	$(DTPA)_2$-anti-MMR	$(DTPA)_3$-anti-MMR	Ladder

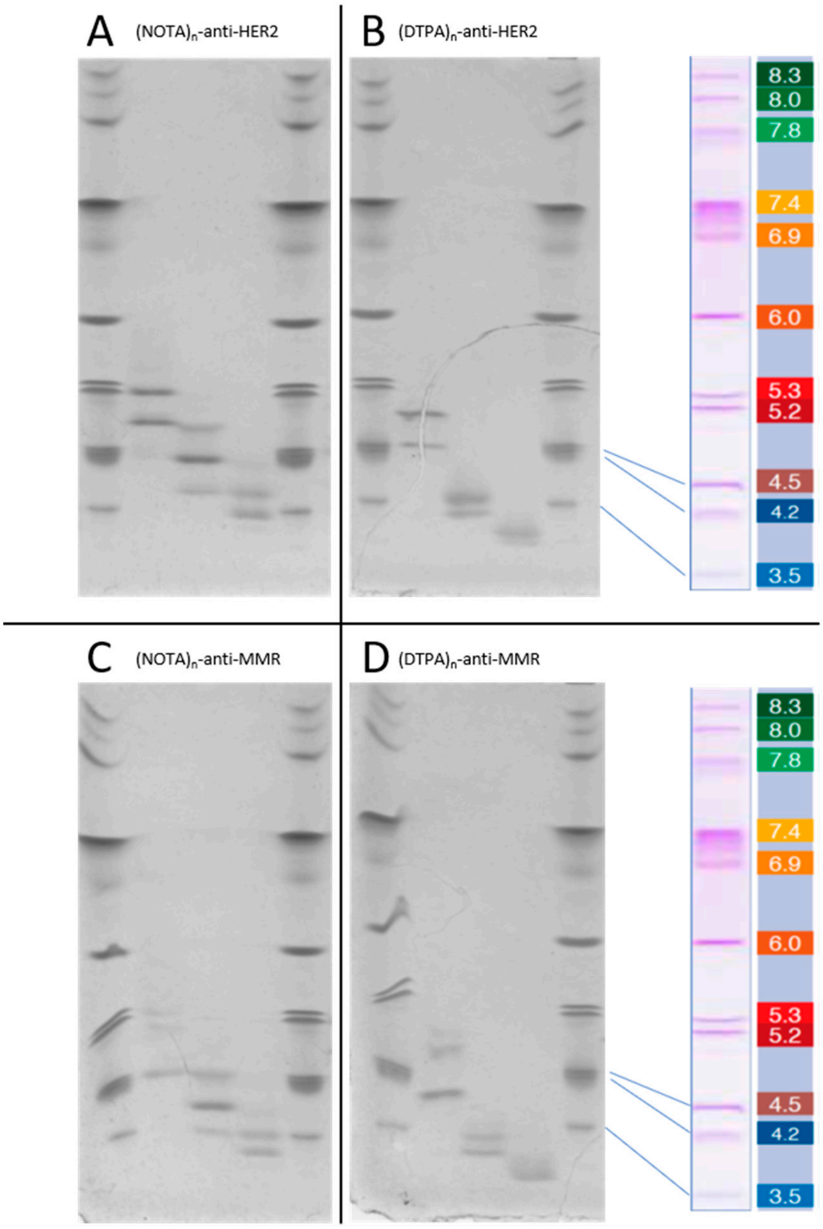

Figure S5. Imaging Iso-electric focusing gel electrophoresis.

Ex vivo biodistribution data

Table S5. Ex vivo biodistribution data [^{68}Ga]Ga-NOTA-anti-HER2.

**Organs**	**[^{68}Ga]Ga-NOTA-anti-HER2**					
	%IA/g			%IA		
	F1	F2	F3	F1	F2	F3
Blood	0.10, σ = 0.04	0.17, σ = 0.14	0.11, σ = 0.05	0.06, σ = 0.02	0.13, σ = 0.17	0.08, σ = 0.07
Heart	0.07, σ = 0.02	0.08, σ = 0.06	0.07, σ = 0.01	0.01, σ = 0.00	0.01, σ = 0.01	0.01, σ = 0.00
Lungs	0.33, σ = 0.04	0.21, σ = 0.08	0.24, σ = 0.11	0.06, σ = 0.01	0.04, σ = 0.02	0.04, σ = 0.01
Liver	0.30, σ = 0.10	0.19, σ = 0.04	0.29, σ = 0.09	0.44, σ = 0.12	0.30, σ = 0.07	0.42, σ = 0.13
Spleen	0.14, σ = 0.06	0.09, σ = 0.02	0.12, σ = 0.05	0.03, σ = 0.01	0.02, σ = 0.00	0.02, σ = 0.01
Pancreas	0.15, σ = 0.04	0.12, σ = 0.05	0.11, σ = 0.04	0.03, σ = 0.01	0.02, σ = 0.00	0.02, σ = 0.01
Kidneys	53.07, σ = 6.31	34.21, σ = 1.56	20.19, σ = 2.58	11.76, σ = 1.16	7.62, σ = 0.44	4.95, σ = 0.33
Stomach	0.18, σ = 0.04	0.22, σ = 0.14	0.63, σ = 1.12	0.05, σ = 0.01	0.04, σ = 0.02	0.19, σ = 0.34
Small Intestine	0.10, σ = 0.02	0.13, σ = 0.07	0.43, σ = 0.74	0.02, σ = 0.00	0.03, σ = 0.02	0.08, σ = 0.15
Large Intestine	0.11, σ = 0.02	0.11, σ = 0.06	0.99, σ = 1.94	0.01, σ = 0.01	0.02, σ = 0.01	0.24, σ = 0.50
Muscle	0.07, σ = 0.02	0.07, σ = 0.05	0.10, σ = 0.12	0.01, σ = 0.00	0.01, σ = 0.00	0.02, σ = 0.02
Tumor	2.68, σ = 0.61	2.39, σ = 1.57	4.30, σ = 2.13	0.97, σ = 0.90	0.06, σ = 0.08	0.36, σ = 0.30
Bone	0.17, σ = 0.06	0.13, σ = 0.08	0.13, σ = 0.05	0.01, σ = 0.01	0.00, σ = 0.00	0.00, σ = 0.00
Lymph nodes	0.35, σ = 0.23	0.29, σ = 0.24	0.27, σ = 0.14	0.01, σ = 0.00	0.00, σ = 0.00	0.01, σ = 0.00

Table S6. Ex vivo biodistribution data [^{111}In]In-DTPA-anti-HER2.

**Organs**	**[^{111}In]In-DTPA-anti-HER2**					
	%IA/g			%IA		
	F1	F2	F3	F1	F2	F3
Blood	0.45, σ = 0.43	0.39, σ = 0.13	0.43, σ = 0.10	0.04, σ = 0.02	0.11, σ = 0.09	0.09, σ = 0.05
Heart	0.21, σ = 0.15	0.21, σ = 0.04	0.24, σ = 0.05	0.02, σ = 0.00	0.03, σ = 0.00	0.03, σ = 0.01
Lungs	0.72, σ = 0.33	0.64, σ = 0.15	0.81, σ = 0.22	0.08, σ =0.02	0.10, σ =0.03	0.14, σ = 0.06
Liver	0.36, σ = 0.09	0.45, σ = 0.09	0.73, σ = 0.09	0.37, σ = 0.06	0.49, σ = 0.09	0.83, σ = 0.12
Spleen	0.18, σ = 0.06	0.24, σ = 0.06	0.34, σ = 0.07	0.02, σ = 0.00	0.03, σ = 0.01	0.05, σ = 0.01
Pancreas	0.13, σ = 0.07	0.14, σ = 0.06	0.21, σ = 0.09	0.01, σ = 0.01	0.01, σ = 0.01	0.03, σ = 0.01
Kidneys	87.94, σ = 8.37	76.96, σ = 7.22	47.66, σ = 7.78	13.24, σ = 0.36	10.55, σ = 4.69	8.01, σ = 0.97
Stomach	0.26, σ = 0.16	0.22, σ = 0.05	0.25, σ = 0.07	0.07, σ = 0.06	0.04, σ =0.01	0.05, σ = 0.01
Small intestine	0.26, σ = 0.16	0.21, σ = 0.09	0.38, σ = 0.24	0.02, σ = 0.01	0.02, σ = 0.01	0.04, σ = 0.04
Large intestine	0.34, σ = 0.18	0.21, σ = 0.10	0.21, σ = 0.03	0.06, σ = 0.05	0.02, σ = 0.01	0.04, σ = 0.02
Muscle	0.23, σ = 0.24	0.14, σ = 0.05	0.30, σ = 0.13	0.02, σ = 0.02	0.02, σ = 0.01	0.03, σ = 0.02
Tumor	9.61, σ = 3.23	8.07, σ = 1.76	6.58, σ = 2.85	0.34, σ = 0.44	0.29, σ = 0.20	0.34, σ = 0.43
Bone	0.19, σ = 0.05	0.33, σ = 0.10	0.40, σ = 0.11	0.01, σ = 0.01	0.01, σ = 0.01	0.02, σ = 0.01
Lymph nodes	0.35, σ = 0.20	0.19, σ = 0.05	0.84, σ = 0.47	0.01, σ = 0.00	0.01, σ = 0.00	0.01, σ = 0.01

Table S7. Ex vivo biodistribution data [^{68}Ga]Ga-NOTA-anti-MMR.

**Organs**	**[^{68}Ga]Ga-NOTA-anti-MMR**					
	%IA/g			%IA		
	F1	F2	F3	F1	F2	F3
Blood	0.64, σ = 0.05	0.51, σ = 0.04	0.61, σ = 0.1	0.12, σ = 0.02	0.14, σ = 0.07	0.19, σ = 0.16
Thymus	1.68, σ = 0.18	1.29, σ = 0.22	0.72, σ = 0.1	0.11, σ = 0.01	0.05, σ = 0.00	0.06, σ = 0.03
Heart	2.14, σ = 0.20	1.92, σ = 0.20	1.14, σ = 0.3	0.27, σ = 0.05	0.20, σ = 0.03	0.13, σ = 0.03
Lungs	1.75, σ = 0.26	1.58, σ = 0.13	1.36, σ = 0.5	0.23, σ = 0.03	0.22, σ = 0.04	0.20, σ = 0.07
Liver	6.15, σ = 1.85	6.70, σ = 1.05	3.62, σ = 0.5	5.19, σ = 0.55	4.32, σ = 0.82	2.87, σ = 0.92
Spleen	4.88, σ = 0.76	3.27, σ = 0.58	1.72, σ = 0.0	0.43, σ = 0.16	0.33, σ = 0.12	0.15, σ = 0.02
Pancreas	1.90, σ = 0.06	1.63, σ = 0.16	0.80, σ = 0.0	0.17, σ = 0.03	0.20, σ = 0.06	0.08, σ = 0.01
Kidneys	36.86, σ = 4.41	50.83, σ = 9.48	43.85, σ = 2.2	5.35, σ = 0.41	7.22, σ = 0.59	5.95, σ = 0.56
Stomach	0.92, σ = 0.30	1.26, σ = 0.71	1.44, σ = 0.1	0.44, σ = 0.02	0.57, σ = 0.26	0.53, σ = 0.14
Small Intestine	0.99, σ = 0.26	0.84, σ = 0.34	0.63, σ = 0.3	0.21, σ = 0.07	0.13, σ = 0.02	0.12, σ = 0.05
Large Intestine	1.09, σ = 0.66	1.53, σ = 0.46	0.76, σ = 0.4	0.40, σ = 0.26	0.50, σ = 0.02	0.18, σ = 0.08
Muscle	0.76, σ = 0.06	0.71, σ = 0.09	0.60, σ = 0.1	0.09, σ = 0.05	0.06, σ = 0.02	0.11, σ = 0.06
Tumor	2.41, σ = 0.09	2.29, σ = 1.04	1.88, σ = 0.3	0.79, σ = 0.74	0.44, σ = 0.32	0.23, σ = 0.15
Bone	2.19, σ = 0.37	1.57, σ = 0.21	0.94, σ = 0.3	0.05, σ = 0.02	0.02, σ = 0.01	0.03, σ = 0.02
Lymph nodes	2.59, σ = 0.35	2.38, σ = 0.16	2.19, σ = 0.8	0.08, σ = 0.02	0.05, σ = 0.02	0.06, σ = 0.03

Table S8. Ex vivo biodistribution data [^{111}In]In-DTPA-anti-MMR.

**Organs**	**[^{111}In]In-DTPA-anti-MMR**					
	%IA/g			%IA		
	F1	F2	F3	F1	F2	F3
Blood	0.48, σ = 0.04	0.56, σ = 0.13	0.63, σ = 0.07	0.20, σ = 0.02	0.22, σ = 0.09	0.11, σ = 0.03
Thymus	1.62, σ = 0.24	1.17, σ = 0.15	0.75, σ = 0.10	0.06, σ = 0.02	0.06, σ = 0.04	0.05, σ = 0.01
Heart	2.09, σ = 0.33	1.36, σ = 0.15	0.96, σ = 0.07	0.24, σ =0.02	0.16, σ =0.01	0.10, σ = 0.01
Lungs	1.64, σ = 0.17	1.18, σ = 0.14	1.05, σ = 0.04	0.27, σ = 0.04	0.20, σ = 0.04	0.19, σ = 0.01
Liver	12.16, σ = 2.12	5.78, σ = 0.88	3.91, σ = 0.59	9.90, σ = 2.94	4.73, σ = 0.92	2.78, σ = 0.64
Spleen	6.26, σ = 0.73	2.89, σ = 0.90	1.87, σ = 0.16	0.61, σ = 0.10	0.30, σ = 0.12	0.20, σ = 0.4
Pancreas	1.99, σ = 0.27	1.48, σ = 1.07	0.79, σ = 0.08	0.14, σ = 0.07	0.12, σ = 0.09	0.05, σ = 0.02
Kidneys	121.44, σ = 12.50	117.28, σ = 16.26	167.75, σ = 16.52	16.04, σ = 1.40	15.24, σ =0.98	21.20, σ = 1.07
Stomach	2.39, σ = 0.30	1.87, σ = 0.27	1.16, σ = 0.06	0.39, σ = 0.03	0.29, σ = 0.04	0.16, σ = 0.04
Small intestine	1.35, σ = 0.21	0.94, σ = 0.18	0.61, σ = 0.07	0.14, σ = 0.02	0.09, σ = 0.07	0.08, σ = 0.03
Large intestine	3.41, σ = 2.05	2.08, σ = 1.32	1.38, σ = 0.55	0.33, σ = 0.21	0.18, σ = 0.09	0.16, σ = 0.02
Muscle	0.95, σ = 0.41	0.80, σ = 0.19	0.47, σ = 0.08	0.11, σ = 0.05	0.10, σ = 0.03	0.06, σ = 0.04
Tumor	3.38, σ = 1.90	2.87, σ = 0.47	1.24, σ = 0.95	0.42, σ = 0.21	0.68, σ = 0.39	0.56, σ = 0.38
Bone	2.17, σ = 0.32	1.44, σ = 0.31	1.45, σ = 0.28	0.05, σ = 0.02	0.02, σ = 0.02	0.02, σ = 0.01
Lymph nodes	3.22, σ = 2.56	2.75, σ = 2.19	0.62, σ = 0.41	0.04, σ = 0.02	0.02, σ = 0.01	0.01, σ = 0.00

Table S9. Ex vivo biodistribution data [^{68}Ga]Ga-NOTA-anti-MMR in KO mice.

**Organs**	**[^{68}Ga]Ga-NOTA-anti-MMR in KO mice**					
	%IA/g			%IA		
	F1	F2	F3	F1	F2	F3
Blood	0.51, σ = 0.13	0.51, σ = 0.06	0.52, σ = 0.03	0.17, σ = 0.06	0.08, σ = 0.01	0.14, σ = 0.07
Thymus	0.16, σ = 0.05	0.19, σ = 0.04	0.21, σ = 0.07	0.01, σ = 0.01	0.01, σ = 0.01	0.01, σ = 0.01
Heart	0.22, σ = 0.06	0.20, σ = 0.01	0.21, σ = 0.05	0.03, σ = 0.01	0.02, σ = 0.00	0.02, σ = 0.01
Lungs	0.38, σ = 0.08	0.40, σ = 0.04	0.53, σ = 0.27	0.08, σ = 0.03	0.07, σ = 0.03	0.09, σ = 0.05
Liver	0.30, σ = 0.03	0.31, σ = 0.02	0.39, σ = 0.05	0.34, σ = 0.11	0.35, σ = 0.09	0.46, σ = 0.07
Spleen	0.19, σ = 0.05	0.20, σ = 0.02	0.23, σ = 0.03	0.02, σ = 0.01	0.02, σ = 0.00	0.02, σ = 0.00
Pancreas	0.12, σ = 0.03	0.16, σ = 0.03	0.16, σ = 0.03	0.02, σ = 0.00	0.01, σ = 0.00	0.02, σ = 0.01
Kidneys	118.97, σ = 27.18	111.33, σ = 10.02	91.22, σ = 7.44	18.68, σ = 3.84	15.36, σ = 1.91	11.95, σ = 1.33
Stomach	0.21, σ = 0.09	0.52, σ = 0.35	0.46, σ = 0.20	0.05, σ = 0.02	0.11, σ = 0.08	0.25, σ = 0.33
Small intestine	0.19, σ = 0.05	0.28, σ = 0.05	0.24, σ = 0.07	0.04, σ = 0.02	0.04, σ = 0.02	0.03, σ = 0.01
Large intestine	0.34, σ = 0.25	0.28, σ = 0.06	0.37, σ = 0.19	0.04, σ = 0.02	0.03, σ = 0.01	0.04, σ = 0.02
Muscle	0.14, σ = 0.06	0.14, σ = 0.03	0.13, σ = 0.04	0.02, σ = 0.01	0.02, σ = 0.01	0.02, σ = 0.01
Bone	0.13, σ = 0.06	0.15, σ = 0.05	0.21, σ = 0.16	0.01, σ = 0.01	0.01, σ = 0.00	0.01, σ = 0.01
Lymph nodes	0.21, σ = 0.07	0.30, σ = 0.04	0.37, σ = 0.20	0.00, σ = 0.00	0.00, σ = 0.00	0.00, σ = 0.00
